# Clinical and CT sialography findings in 22 dogs with surgically confirmed sialoceles

**DOI:** 10.1111/vru.13104

**Published:** 2022-05-30

**Authors:** Yi Lin Tan, Ana Marques, Tobias Schwarz, Jordan Mitchell, Tiziana Liuti

**Affiliations:** ^1^ Royal (Dick) School of Veterinary Studies and Roslin Institute The University of Edinburgh Roslin UK; ^2^ Vets Now Emergency and Specialty Hospital Glasgow UK

**Keywords:** CT sialography, dog, salivary gland, salivary mucocele

## Abstract

Sialoceles are an uncommon canine salivary gland disease, and complete surgical resection is important for a positive outcome. Radiographic sialography has been described as a diagnostic test for presurgical planning; however, superimposition artifacts may limit the diagnosis and detection of all affected glands. Computed tomographic (CT) sialography is a promising technique for delineating the salivary gland apparatus. The aims of this retrospective, observational study were to describe clinical and CT sialographic findings in a group of dogs with confirmed sialoceles, to determine the sensitivity of CT sialography for detecting affected salivary glands using surgery as the reference standard and to determine interobserver agreement for CT sialographic assessments. Dogs were included if they underwent a CT sialography study followed by surgical resection of the diseased gland(s) and histopathological analysis. Computed tomography sialography studies of dogs with surgically confirmed sialoceles (n = 22) were reviewed by a European College of Veterinary Diagnostic Imaging (ECVDI)‐certified radiologist and an ECVDI resident. Interobserver agreement was calculated using Cohen's kappa statistics. CT sialography results were compared to surgical findings to determine sensitivity. Contrast leakage was detected in 12 of 22 dogs (54.5%), with intrasialocele leakage being most frequently observed (7/12, 58.3%). There was substantial agreement (κ = 0.70) between reviewers identifying diseased glands, substantial agreement (κ = 0.62) on the diagnostic quality, and no to slight agreement (к = 0.13) in the detection of contrast leakage. The overall sensitivity of CT sialography to detect surgically confirmed diseased glands was 66.7% (95% confidence interval: 48.8‐80.8). In conclusion, these findings support the use of CT sialography as an adjunct diagnostic test for treatment planning in dogs with sialoceles.

**Abbreviations**
ECVDI: European College of Veterinary Diagnostic Imaging
ECVS: European College of Veterinary Surgeons

## 1 INTRODUCTION

Salivary gland pathology is relatively uncommon in dogs and cats, with a reported overall incidence of less than 0.3 percent.^1^, ^2^ Salivary gland disease includes sialoceles, also known as salivary mucoceles, sialolithiasis, idiopathic and phenobarbital‐responsive sialadenosis,^3^, ^4^, ^5^ sialadenitis, abscessation and neoplasia,^1^ with specific salivary gland pathology including parotid sialoceles,^6^ nasopharyngeal sialoceles,^7^ zygomatic sialadenitis^8^ and salivary gland neoplasia^9^, ^10^, ^11^ described in the current literature. Sialocele formation is one of the most common presentations of salivary gland pathology, representing 11% of a large cohort of salivary gland samples submitted for histopathology,^1^ and is defined as submucosal or subcutaneous accumulation of saliva within a fibrous capsule.^12^, ^13^, ^14^ In dogs, the most commonly affected gland is the monostomatic sublingual gland,^13^, ^15^, ^16^, ^17^, ^18^, ^19^, ^20^ giving rise to the characteristic ventral neck swelling and ranula formation.^21^ Underlying causes of sialoceles have been suggested to include trauma, foreign bodies, sialolithiasis, and obstruction of the duct secondary to inflammation. Idiopathic sialocele formation, where an underlying cause has not been identified, has been more commonly reported.^12^, ^13^, ^16^, ^22^, ^23^, ^24^, ^25^ The salivary apparatus of the dog is complex, and surgical management of salivary gland pathology can be challenging due to the close proximity to neurovascular bundles, lymph nodes and lymphatic channels.^23^ Presurgical identification of the correct diseased gland and respective ducts is important as curative treatment for sialoceles entails sialoadenectomy, i.e., complete surgical resection of the affected salivary gland and ligation of the respective salivary duct.^12^, ^13^, ^16^ Recurrence has been reported^27^ if complete resection is not achieved.^15^, ^16^, ^23^, ^27^

Various imaging modalities and techniques have been described to characterise the salivary gland apparatus and aid in the diagnosis of salivary gland disease. This includes conventional radiography,^28^, ^29^ contrast radiography or “sialography”,^13^, ^26^ ultrasound^30^, ^31^ and computed tomography (CT).^31^, ^32^, ^33^, ^34^, ^35^ Radiographic sialography has been described to aid presurgical planning in canine sialoceles.^13^, ^26^ However, with radiographic examinations of the head and skull, diagnosis is challenging due to organ superimposition. Use of CT as part of a diagnostic work‐up has been reported for zygomatic mucoceles,^31^, ^32^, ^33^ zygomatic sialolithiasis^34^ and parotid duct foreign bodies,^35^ reporting advantages of cross‐sectional imaging in the assessment of the salivary gland apparatus. A previous cadaver CT sialography study that described the anatomy of the parotid, mandibular and zygomatic salivary glands demonstrated the valuable information CT sialography provides in the assessment of the salivary gland.^36^ A recent study described the CT features of sialoceles;^37^ however, sialography was not performed in these cases. Based on a current literature search, there is no study describing CT sialography features of surgically confirmed sialoceles or assessing the sensitivity of CT sialography in determining the diseased gland of origin.

Therefore, the aims of the current study were threefold: to first describe the CT sialography features in dogs with surgically confirmed sialoceles; to evaluate the diagnostic quality of the sialography studies and recognition of contrast leakage; and last, to determine the sensitivity of CT sialography in the identification of the diseased salivary gland of origin compared to surgery, taken to be the reference standard test. We hypothesized that there would be strong agreement between reviewers in the identification of diseased glands on CT sialography, evaluation of the diagnostic quality of the study, and recognition of contrast leakage. Additionally, we hypothesized that CT sialography would be a sensitive technique to identify the diseased salivary gland of origin.

## 2 MATERIALS AND METHODS

### 2.1 Case selection

This was a retrospective single‐institutional observational study. Ethical approval was obtained from the institution's ethical review committee (Veterinary Ethical Review Committee) prior to the collection of retrospective data and the commencement of this study (50.21). Medical records at the University of Edinburgh, Royal (Dick) School of Veterinary Studies, Hospital for Small Animals (HfSA), were searched for client‐owned dogs with surgically confirmed sialoceles between 2011 and 2019. Key words used to search included “sialocele”, “salivary mucocele” and “ranula”. Dogs were included if they had surgically confirmed sialoceles following a CT sialography study and if they had histopathological reports available for review. Patients were excluded if they did not undergo a CT sialography study or surgical exploration and if full surgical and histopathological records were not available. The presenting clinical signs and signalment, including weight, age, sex, neuter status, and breed, were tabulated from the medical records. Additionally, the following data were extracted from the medical records: first, complete CT reports detailing CT sialography findings, gland(s) cannulated and final imaging diagnoses; second, full surgical notes including the surgical approach, intraoperative gross findings as well as affected gland(s) resected and last, final histopathological reports with confirmed histopathological diagnoses. Histopathological analysis was performed on submitted partial biopsied specimens or complete resected glands. Final inclusion of cases was determined by consensus between a European College of Veterinary Surgeons (ECVS)‐certified surgeon (A.M.), European College of Veterinary Diagnostic Imaging (ECVDI)‐certified radiologist (T.L.), and a second year ECVDI diagnostic imaging resident in training (Y.L.T.).

### 2.2 Image analysis

A standardized soft tissue window was used for analysis (window width 350 HU, window level 40 HU). All images were reviewed on a computer workstation (Apple Mac Pro, Apple, USA) with a calibrated LCD flat screen monitor using dedicated DICOM viewer software (Horos, Purview, Annapolis MD, USA, version 3.3.6) independently by an ECVDI‐certified radiologist (T.L.) and a second year ECVDI diagnostic imaging resident in training (Y.L.T.). All studies were randomized and anonymized prior to analysis. Precontrast and sialography studies were evaluated in all cases. If available, the IV postcontrast studies were evaluated in conjunction with the precontrast series. Both reviewers were aware that all cases had confirmed sialoceles. Both reviewers were unaware of patient signalment, presenting clinical signs, CT sialography, surgical findings and histopathological results. Standardized locations for specific measurements of HU and thickness were established prior to analysis. In the incidence of any disagreement, final findings were agreed upon by consensus between the two reviewers. The precontrast and CT sialography followed by postcontrast studies (where available) were evaluated sequentially for the affected gland of origin and laterality. Pre‐ and postcontrast (where available) CT studies were evaluated in conjunction for the following criteria: affected salivary gland size (atrophied, normal, enlarged), salivary gland density (fatty infiltration [−100 to −20 HU], fluid pockets [0 to 20 HU], edematous, normal soft tissue, soft tissue mass, mineralized [>100 HU]), accumulation of nonenhancing low‐density fluid [0 to 20 HU] and measurement of fluid density in HU, presence or absence of mineralization, presence of absence of a capsule (and where present, capsule thickness), local soft tissue appearance (normal, fat stranding, localized swelling), and assessment of the lymph nodes of the head, namely, the parotid, mandibular, medial retropharyngeal and where present, the lateral retropharyngeal lymph nodes. The size of these regional lymph nodes was assessed qualitatively based on nodal width as well as on asymmetry. Lymph nodes were classified as normal or enlarged. Specifically, in the available IV postcontrast studies, the presence or absence of sialocele contrast enhancement, as well as the distribution of contrast enhancement, was recorded. The diagnostic quality of the CT sialography studies was assessed and graded as follows: nondiagnostic, poor (partial filling of duct), fair (partial filling of duct and gland) and good (complete filling of the duct and gland). The cannulated duct and gland were assessed for the presence and location (periductal, peri‐glandular, or intrasialocele) of contrast medium leakage.

### 2.3 Statistical analysis

Statistical tests were selected by three authors (Y.L.T., J.M., T.L.) and performed by a PhD student (J.M.) with experience in statistical analysis of diagnostic tests who had also completed an institutional course in statistics and study design. Statistical software was used (Minitab 17, Minitab Inc., State College, PA and GraphPad Prism 9, GraphPad Software, Inc. La Jolla, CA). Statistical significance was defined as *P *< 0.05. Interobserver agreement was assessed using Cohen's kappa for identification of the diseased gland(s) on CT sialography, diagnostic quality scores and the presence of contrast leakage. The overall sensitivity of CT sialography in detecting the diseased gland against surgery as the reference standard was calculated. Additionally, the sensitivity of CT sialography in detecting the correct gland was calculated for each gland where appropriate. Specificity was not calculated in the absence of a true negative population in the study. Summary statistics (mean, median and range) were calculated for signalment data, namely, weight and age.

## 3 RESULTS

### 3.1 Population

**FIGURE 1:**
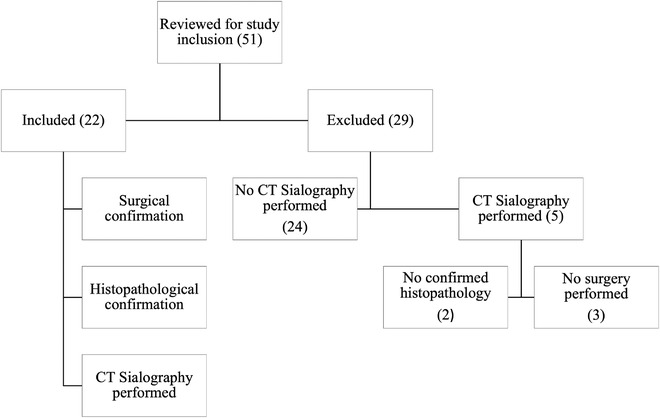
Selection criteria and exclusion process for the final study population

A total of 51 confirmed cases of sialoceles were retrieved from the HfSA database, with 22 dogs meeting the inclusion criteria. Figure 1 summarizes the selection process that was used for the study population. The study population comprised eight neutered males, seven entire males, five neutered females and two entire females. There were three Cocker Spaniels (3/22; 13.6%), three Border Collies (3/22; 13.6%), two Siberian Huskies (2/22; 9.1%), two Labrador Retrievers (2/22; 9.1%), two Staffordshire Bull Terriers (2/22; 9.1%), three mixed‐breeds (3/22; 13.6%), and one of the following breeds: Miniature Dachshund, Bearded Collie, Beagle, Northern Inuit dog, Lhasa Apso, Cavalier King Charles Spaniel, and Collie. The mean body weight was 19.5 kg, with a median of 17.9 kg (range: 8.0–41.8 kg). The mean age was 4.5 years, and the median age was 3.6 years (range 0.3–12.4 years). The most common clinical sign was a mass or swelling in the mandibular region (11/22, 50.0%), and three of the 22 dogs (13.6%) displayed a combination of two or more of the above clinical signs. Table 2 summarizes the presenting clinical signs. All cases had surgical intervention either on the day or within 24‐48 h post‐CT sialography except one case that was initially medically managed following diagnosis on the initial CT sialography study. This case represented 13 days after failure of medical management, and a repeat CT sialography study was performed to include the contralateral side for presurgical planning and comparison. Surgery was performed on the same day as the repeated CT sialography. The initial CT sialography study was assessed for this case, as diagnosis was based on the initial study, and a complete head and neck study including pre‐ and postcontrast examinations was available for evaluation.

### 3.2 CT sialography protocol

Standardized positioning and head and neck CT studies based on routine standardized protocols were performed on all patients. All patients were positioned in sternal recumbency within a foam trough with their heads positioned on a separate foam support. A complete CT sialography protocol was performed for all included patients, which comprised a precontrast series and a CT sialography series. Additionally, if performed, an intravenous (IV) postcontrast series following a standardized protocol^38^ was acquired either before or after the CT sialography study. The order in which this was performed varied depending on the surgeon's preference for the operator and soft tissue. All patients who underwent an IV postcontrast administration study received a standardized dose of 2 ml/kg iodinated contrast medium; for cases from 2011 to October 2016, iopamidol (Niopam^®^ 340, Bracco UK Ltd) was used, and for cases after October 2016, ioversol (Optiray^®^ 350, Guerbet, France) was used. A fixed‐duration delay of 40 seconds was implemented for dogs up to 20 kg, and 50 seconds for dogs above 20 kg. Images were acquired using a 4‐row MDCT unit (Somatom^®^ Volume Zoom, Siemens, Germany) for the 19 cases presenting from 2011 to October 2016 and subsequently a 64‐row MDCT scanner (Somatom^®^ Definition AS Siemens, Erlangen, Germany) for the three cases presenting from October 2016 to September 2019.

The following image acquisition parameters were used: slice thickness 2.0 mm, pitch between 1 and 1.5, tube potential 120 kVp, matrix 512×512 (cases presenting from 2011 to October 2016); slice thickness 1.0 mm, pitch 0.55, tube potential 120 kVp, matrix 512×512 (cases since October 2016). For both scanners, application of automated dose control resulted in variable tube current depending on the size of the patient. Studies were undertaken with a standardized, optimized, protocol for the head. Soft tissue and bone kernel (Siemens proprietary H40 and H70, respectively) reconstruction images with matching bone and soft tissue windows were used.

The suspected diseased salivary gland(s) were identified based on the location and distribution of the sialocele on the precontrast CT images and presenting clinical examination findings. Specific clinical features and precontrast CT characteristics were used to determine the gland of interest. The CT characteristics are summarized in Table 1 and were used in combination with the clinical features as follows:

**TABLE 1:** Precontrast CT characteristics used to determine the suspected gland of origin

Suspected diseased gland of origin	Precontrast CT characteristics
Sublingual	Ranula formation, fluid‐attenuating accumulation between the caudal level of the mandible and tongue ± extension into the ventral cervical subcutaneous tissues. Normal gland small and difficult to delineate.
Mandibular	Extension of fluid‐attenuating accumulation ventrolateral/ventromedial to the mandibular gland (normally located caudal to the mandibular ramus). ± Extension within the ventral cervical subcutaneous tissues. Any change in normal oval shape or size.
Parotid	Fluid‐attenuating accumulation within the subcutaneous tissues surrounding the external ear canal and dorsal to the ipsilateral mandibular salivary gland. Any change in normal crescent shape and size.
Zygomatic	Fluid‐attenuating accumulation within the pterygopalatine fossa, lateral to the origin of the pterygoid muscle. Any change in normal triangular shape and size.

**TABLE 2:** Clinical signs for 22 dogs with surgically confirmed sialoceles

Presenting Clinical signs	NUMBER (%)
Mandibular Swelling/Mass	11 (50)
Neck swelling/Mass	6 (27.3)
Sublingual Swelling	8 (31.8)
Exopthalmos	1 (4.5)

^*^Total number exceeds 22 as three dogs displayed a combination of two or more different clinical signs.

First, for submandibular or cervical lateralized swellings (cases 1–2, 5–6, 8–18), in cases where a ranula was present (cases 2, 9, 12–16), the aim was to first cannulate the sublingual duct, as the sublingual salivary gland is the most commonly affected.^13^, ^15^, ^16^, ^17^, ^18^, ^19^, ^20^, ^21^ In the absence of a ranula (cases 1, 5–6, 8, 10–11, 17–18) and when only one of the sublingual caruncle openings was visible to the operator, the visible duct opening was cannulated, and contrast medium was injected. Depending on whether the sublingual or mandibular gland was cannulated initially and if the corresponding sialogram was normal, the cannula was then retracted proximally, and contrast medium was injected to attempt uptake of contrast medium by the other salivary duct and respective gland. For nonlateralized cervical swelling (cases 3–4, 7, 20–22), the suspected salivary duct caruncle was cannulated, and contrast medium was injected. If the sialogram appeared unremarkable, cannulation of the contralateral duct was considered to help determine which submandibular salivary gland complex was affected (right or left). This technique (case 6) was also used where uptake of contrast medium by the gland was present, but the attenuation of the glandular tissue appeared abnormal, and comparison with the contralateral side helped determine if disease was present. For patients with exophthalmos (case 19), the corresponding zygomatic duct was cannulated after identifying the respective ipsilateral zygomatic caruncle at the caudal aspect of the last upper molar tooth. For caudal mandibular or mandibular swellings that extended into the face and upper lip (cases 6 and 17), the ipsilateral parotid duct was cannulated after identification of the caruncle at the level of the maxillary fourth premolar tooth.

The suspected duct of interest was grossly identified in accordance with its anatomic description in the standard literature^39^ as well as based on findings from a previous unpublished CT sialography cadaveric study performed at this institution, where ducts of their corresponding salivary glands were cannulated and injected with methylene blue and contrast media, followed by CT. The duct of interest was cannulated with an 18–25G cannula (Becton Dickinson, Wokingham, UK), and diluted iodine‐based, nonionic contrast medium (Iopamidol, Niopam^®^340, Bracco Ltd, UK; Ioversol, Optiray^®^ 350, Guerbet, France, concentration of 180 mg iodine/ml) was injected. The volume injected varied according to body weight, and the gland catheterized until resistance was felt or backflow of contrast through the catheter was observed. The specific range of volume for each gland was as follows: zygomatic salivary gland 1–2 ml, mandibular salivary gland 1–5 ml, sublingual salivary gland 1–5 ml, and parotid salivary gland 1–5 ml. Images were immediately acquired following this procedure under the same protocol as for the precontrast series.

### 3.3 Surgical findings and histopathology report analysis

Surgical exploration of the suspected diseased gland was performed. A ventral approach with tunnelling under the digastricus muscle^12^ was the approach used for sublingual or mandibular salivary glands (“submandibular complex”), a lateral approach without zygomatic arch ostectomy was adopted for the zygomatic gland, and a lateral approach with a longitudinal incision ventral to the external acoustic meatus was used for the parotid salivary glands.^23^ Final histopathological diagnoses were extracted from the final diagnoses made in the report. If present, additional comments were also reviewed to confirm the final histopathological diagnoses. Sialoceles were defined as the accumulation of salivary secretions in single or multiloculated cavities not lined by secretory epithelium, and sialadenitis was defined as inflammation of the salivary glands.^40^

### 3.4 CT sialography findings

Twenty‐three sialoceles were identified in 22 dogs. Fourteen sialoceles were left‐sided, and nine were right‐sided; one dog had bilateral sialocele formation. Based on CT sialography, 22 diseased glands of origin could be identified from these 23 sialoceles, i.e., one gland of origin could not be identified despite the presence of a sialocele (Supplement 1). In one dog, two diseased glands were identified (bilateral involvement, case 15), whereas in another dog, no diseased gland of origin could be identified (nondiagnostic CT sialography study, case 7). The number of dogs with diseased glands identified on CT sialography was as follows: dogs with sublingual (14/22, 63.6%), mandibular (5/22, 22.7%), parotid (2/22, 9.1%) and zygomatic gland pathology (1/22, 4.5%). No lymphadenomegaly was detected in any dog. Table 3 summarizes the key CT sialography findings.

**TABLE 3:** Key CT sialography findings for 22 dogs with confirmed sialoceles

Feature assessed	Results
Size of affected salivary gland	Normal: 21/22 (95.4%)
	Enlarged: 1/22 (4.5%)
Density of affected salivary gland	Soft tissue dense in 22/22 (100%), Median: Range: 42.0‐65.0 HU
Size of regional lymph nodes	Normal: 22/22 (100%)

**FIGURE 2:**
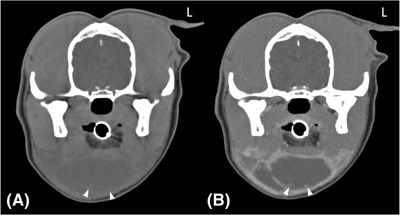
A,B, Transverse precontrast (A), postcontrast (B) CT images (Somatom^®^ Volume Zoom 4‐slice Siemens), soft tissue algorithm (manually windowed to WW = 650, WL = 150), slice thickness 2 mm, kVp 120, mAs 200) of an 8‐year‐old, male entire collie presenting with right submandibular swelling at the level of the temporomandibular joints. Note the ill‐defined contrast‐enhancing nodular protrusions from the sialocele wall (arrowheads) L: Left

**FIGURE 3:**
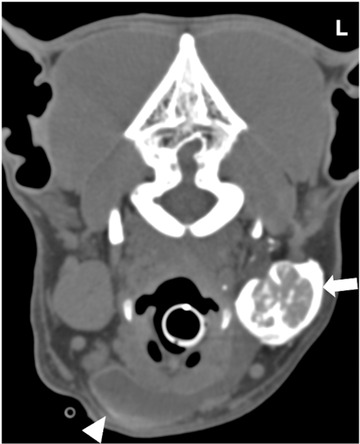
Transverse CT (Somatom^®^ Volume Zoom 4‐slice Siemens), soft tissue algorithm (manually windowed to WW = 750, WL = 150), slice thickness 2 mm, kVp 120, mAs 200) image of a 4‐year‐old male column presenting with ventral neck swelling at the level of the mandibular salivary glands post sialography. Note the contrast‐enhancing left mandibular salivary gland (arrow) and the contrast medium leaking within the sialocele (arrowhead) L: Left

**FIGURE 4:**
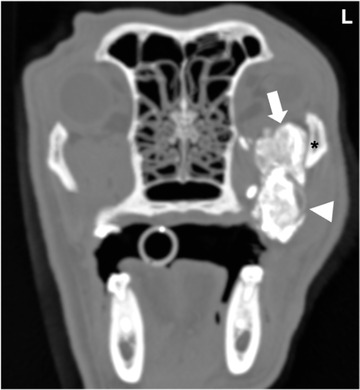
Transverse CT image (Somatom® Volume Zoom 4‐slice Siemens), soft tissue algorithm (manually windowed to WW = 2500, WL = 100), slice thickness 2 mm, kVp 120, mAs 200) of a 10‐year‐old female neutered Labrador retriever presenting with L exophthalmos at the level of the zygomatic salivary gland post sialography. Note the contrast‐enhancing left zygomatic salivary gland (arrow) and the contrast medium leaking within the sialocele (arrowhead). Note the left zygomatic arch (*) L: Left

**FIGURE 5:**
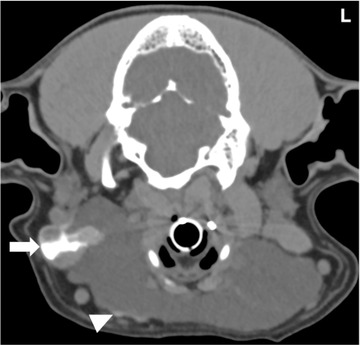
Transverse CT image (Somatom® Definition 64‐slice Siemens), soft tissue algorithm (manually windowed to WW = 750, WL = 150), slice thickness 1 mm, kVp 120, mAs 260) of a five‐year‐old female neutered bearded collie presenting with a fluid‐filled neck mass at the level of the sublingual salivary glands post sialography. Note the contrast‐enhancing right sublingual salivary gland (arrow) and the contrast medium leaking within the sialocele (arrowhead) L: Left

**FIGURE 6:**
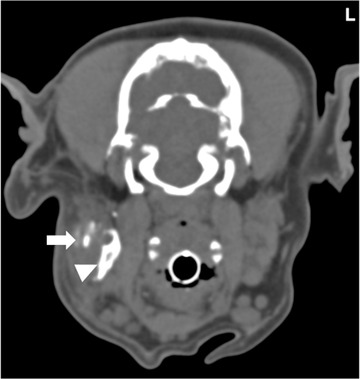
Transverse CT (Somatom^®^ Volume Zoom 4‐slice Siemens), soft tissue algorithm, (manually windowed to WW = 750, WL = 150), slice thickness 2 mm, kVp 120, mAs 200) image of a twelve‐year‐old, male neutered cavalier King Charles spaniel presenting with jaw swelling at the level of the parotid salivary glands post sialography. Note the contrast‐enhancing right parotid salivary gland (arrow) and the contrast medium leaking within the sialocele (arrowhead) L: Left

There was substantial agreement (κ = 0.70) between the two reviewers identifying dogs with diseased glands, with 18/22 (81.8% [95% confidence interval (CI): 59.7‐94.8]) in agreement. CT sialography studies were scored as good in 15/22 dogs (68.2%), fair in 1/22 (4.5%), poor in 5/22 (22.7%) and nondiagnostic in one (4.5%) of the cases. There was substantial agreement (к = 0.62) between the two reviewers on the CT sialography scores, with 19/22 (86.4% [95% CI: 65.1‐97.1]) in agreement. Contrast leakage was detected in 12/22 (54.5%) of the dogs at the following locations: intrasialocele (7/12, 58.3%), periglandular (5/12, 41.6%), and periductal 3/12 (25%). Of these, there was one case of concurrent peri‐glandular and intrasialocele leakage and one case of periductal and intrasialocele leakage. Agreement was absent to slight (к = 0.13) between the reviewers on the presence or absence of contrast leakage, with 12/22 (54.6% [95% CI: 32.2‐75.6]) cases in agreement. Overall, our hypotheses that there would be strong agreement between reviewers in the identification of diseased gland(s) on CT sialography and assessment of the quality of the study were confirmed, but not for the recognition of contrast medium leakage. All 23 identified sialoceles displayed a focal accumulation of nonenhancing fluid (median: 10.3 HU, range: 4.8‐21.5 HU). Capsule formation was detected in 21/23 sialoceles (86.9%), with a median thickness of 1.8 mm and a range of 1.0 ‐ 3.8 mm. Eighteen of the 21 (85.7%) displayed peripheral rim enhancement. One case (case 5) also displayed ill‐defined contrast‐enhancing nodular soft tissue changes that extended from the capsule into the sialocele (Figure 2A‐B). The soft tissues adjacent to the identified sialoceles were normal in 14/23 (60.1%), with fat stranding identified in 9/23 (39.1%). Table 4 summarizes the key CT sialography findings related to the identified sialoceles. Figures 3, 4, 5, 6 ‐ demonstrate CT sialography images of four dogs

**TABLE 4:** CT sialography features related to identified sialoceles (n = 23)

Feature assessed	Results
Accumulation of nonenhancing low‐density fluid ± mineralization	Present: 23/23 (100%), Median: Range: 4.8‐21.5 HU
Capsule formation	21/23 (86.9%),
Rim enhancement of capsule	19/21 (85.7%)
Local soft tissue changes	Normal: 14/23 (60.1%), Fat stranding: 9/23 (39.1%)

### 3.5 Surgical findings

A total of 30 diseased glands were identified intraoperatively. The most affected gland was the sublingual (18/30, 60%), followed by the mandibular (9/30, 30%), parotid (2/30, 6.7%) and zygomatic glands (1/30, 3.3%). Twelve of the 22 dogs had left‐sided glandular involvement, and eight dogs had right‐sided glandular involvement. Two of the 22 cases had bilateral disease: one involving the submandibular gland complex and one involving the right sublingual and contralateral submandibular gland complex.

### 3.6 Histopathology results

Sialocele formation was confirmed in 14 of 22 (63.6%) dogs. Of these, concurrent pathologies extracted from the histopathological reports included sialadenitis (7/14, 50.0%), secondary abscessation (1/14, 7.1%), cellulitis (1/14, 7.1%), and edema (1/14, 7.1%). The remaining four dogs demonstrated sialocele formation as the sole pathology. In seven of 22 (31.8%) cases, sialadenitis was the sole pathology confirmed. In the last case, interstitial hemorrhage was reported.

Sixteen dogs demonstrated inflammatory or infectious pathologies, such as sialadenitis (either as the sole or concurrent pathology), abscessation, and cellulitis. Surrounding edema and fat stranding were detected in six of 16 (37.5%) dogs, and 12 of 16 (75.0%) dogs displayed rim enhancement. In the four dogs where the only finding on histopathology was sialocele formation, three displayed rim enhancement, and fat stranding was identified in two dogs. Supporting Information S1 summarizes the CT sialography, surgical and key histopathological findings of the study population. The clinical signalment, presenting clinical signs and individual CT findings are detailed in Supporting Information S2.

### 3.7 Sensitivity of CT sialography

The overall sensitivity of CT sialography to detect surgically confirmed diseased glands was 66.7% (95% CI: 48.8–80.8). For the sublingual and mandibular glands, the sensitivity was 72.2% (95% CI: 49.1–87.5) and 44.4% (95% CI: 18.9–73.3), respectively. The number of cases of parotid (n = 2) and zygomatic (n = 1) gland disease were too small for individual meaningful statistical analysis. At surgery, involvement of both the sublingual and mandibular glands was identified in five dogs, but retrospective analysis of the CT sialography studies only identified single glands as the gland of origin. An additional case of bilateral sublingual and mandibular gland involvement identified at surgery only displayed evidence of unilateral sialocele formation on CT sialography. In the cases where a single diseased gland was identified at surgery, CT sialography findings differed from the surgical findings in two cases: incorrectly identifying the mandibular gland when the sublingual was affected and vice versa.

**FIGURE 7:**
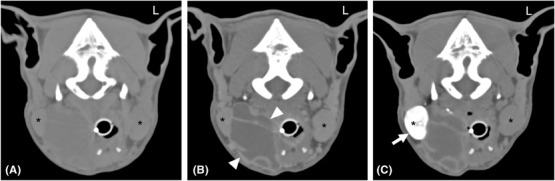
A**‐C** Transverse A, precontrast, B, postcontrast and C, post CT (Somatom^®^ Volume Zoom 4‐slice Siemens), soft tissue algorithm, (manually windowed to WW = 750, WL = 150), slice thickness 2 mm, kVp 120, mAs 100) sialography images of a four‐year‐old male neutered border collie at the level of the mandibular glands, presenting with sublingual and mandibular swelling that extended toward the neck Note the rim enhancement of the sialocele (arrowheads), and normal Rt mandibular sialogram (arrow). A right sublingual sialocele was diagnosed based on the absence of contrast leakage, which was confirmed intraoperatively. Note both the left and right mandibular salivary glands (*) L: Left

**FIGURE 8:**
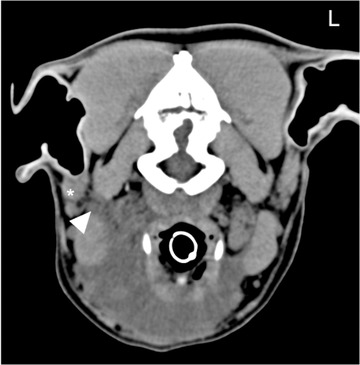
Transverse precontrast CT (Somatom^®^ Volume Zoom 4‐slice Siemens), soft tissue algorithm (manually windowed to WW = 250, WL = 50), slice thickness 2 mm, kVp 120, mAs 200) image of an eight‐year‐old, male entire collie, presenting with right submandibular swelling at the level of the parotid salivary glands. Note the dorsal extension of the encapsulated fluid (arrowhead) ventromedial to the right parotid salivary gland (*) L: Left

Ten cases displayed no contrast medium leakage. In four of these cases, a sublingual sialocele was diagnosed based on a normal mandibular sialogram in combination with previously specified clinical and precontrast CT features. Figure 7A–C depicts one such case. Of these four cases, resistance to contrast medium injection at the sublingual caruncle was found in one case (case 14), suggestive of duct obstruction. In another case (case 5), the surgical swelling and fluid accumulation extended dorsally to the base of the parotid gland, raising concerns regarding the possible involvement of the parotid salivary gland (Figure 8). A parotid sialogram was performed to rule out involvement of this gland in the disease process.

In three other cases where no contrast leakage was detected, the correct gland of origin was correctly identified based on the specific clinical and CT anatomical features demonstrated by these cases. Despite no detectable leakage on a mandibular sialogram, a mandibular sialocele was incorrectly diagnosed where a sublingual sialocele was detected intraoperatively in one case (case 8). CT sialography was nondiagnostic in the last case.

### 3.8 Diagnostic utility of CT sialography

**FIGURE 9:**
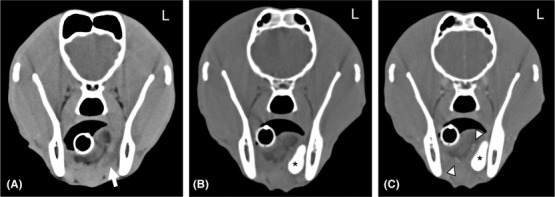
A–C, Transverse (A) precontrast, (B) post sialography and (C) postcontrast CT (Somatom^®^ Volume Zoom 4‐slice Siemens), soft tissue algorithm, (manually windowed to WW = 750, WL = 150), slice thickness 2 mm, kVp 120, mAs 100) images of a two‐year‐old female neutered border collie that presented with swelling under her tongue. Note the encapsulated fluid pocket under the tongue (arrow), contrast leakage into the sialocele (*) and presence of contrast within the vasculature (arrowhead). In this case, the postcontrast study could not be evaluated independently L: Left

Thirteen dogs had CT sialography performed prior to the postcontrast series. One case did not have a postcontrast study. Of the remaining eight cases where CT sialography was performed after the postcontrast study, findings from the CT sialography study differed from those of the postcontrast study in three cases (37.5%). In these cases, CT sialography provided additional information to allow correct identification of the affected gland. Specifically, a consensus could not be reached between the reviewers based on the postcontrast findings in one case, and assessment of the CT sialography study allowed the reviewers to make a confident diagnosis of the affected gland. In the one case where CT sialography was nondiagnostic, a diagnosis was made in the postcontrast study. Objective assessment of the salivary gland apparatus on the postcontrast studies was not possible in the 13 cases where CT sialography was performed prior to the postcontrast study. This was because residual contrast medium within the ducts and/or glands was often present, which hindered unbiased evaluation of the salivary gland apparatus. As a result, diagnosis of the gland of origin based on the postcontrast study was not made in these cases. Figure 9A–C depicts the precontrast, CT sialography and postcontrast images of a case illustrating the presence of intrasialocele leakage on the postcontrast study obtained after CT sialography was performed.

CT sialography prior to the postcontrast study allowed for the correct identification of diseased salivary glands in 12 of 13 (92.3%) cases. In contrast, diagnosis based only on the postcontrast findings prior to CT sialography identified the correct gland in five of eight (62.5%) cases.

## 4 DISCUSSION

This study is the first published report describing CT sialography features as well as the sensitivity of CT sialography in surgically confirmed canine sialoceles. The findings presented in this study supported our hypothesis that there would be strong agreement between reviewers in the identification of diseased glands on CT sialography and evaluation of the diagnostic quality of the study. However, this study did not support our hypothesis that there would be strong agreement for the recognition of contrast leakage, which was detected in 12/22 dogs (54.5%); intrasialocele leakage was most frequently observed (7/12, 58.3%). Additionally, this study found that CT sialography only demonstrated an overall moderate sensitivity of 66.7% (95% CI: 48.8–80.8) in the identification of the diseased salivary gland of origin. The findings presented in this study agree with previous reports highlighting that canine salivary gland disease, and more specifically sialoceles, are rare conditions with only 51 confirmed cases over a period of nine years. The monostomatic sublingual gland was the most affected gland in our study, which concurs with previously reported incidence.^13^, ^15^, ^16^, ^17^, ^18^, ^19^, ^20^ Reported predisposing factors such as sialadenitis were seen in 14 of the 22 dogs in our study. All cases in our study displayed a focal accumulation of nonenhancing low attenuating fluid (median: 10.3 HU), with the majority encapsulated within a thin, soft tissue attenuating, contrast‐enhancing wall. These main findings are in agreement with a recently published study, which also identified intrasialocele contents with a median of 18.5 HU and similar homogeneously contrast‐enhancing walls.^37^ As it was not the primary aim of the present study to describe CT features of sialoceles, not all characteristics highlighted in the previous study, such as the shape and size of the sialoceles, were investigated. Additionally, as our study focused on the identification of the diseased gland of origin, we did not classify the sialoceles according to the CT and surgical characteristics described in the previous study.^37^ Interestingly, in our study, one case (case 5) also demonstrated similar nodular intrasialocele protrusions (Figure 2A,B); however, this was less common than in the previous study, and “frond‐like” protrusions were not observed in the current study.^37^ The results from our study are also in agreement with previous reports, with unilateral involvement more commonly reported than bilateral involvement^12^, ^13^, ^24^. However, in our findings, left‐sided involvement was more frequent than right‐sided involvement, which differed from a previous study.^37^ Adjacent fat stranding was present in nine of the 23 sialoceles (39.1%), which is higher than the two out of the 13 sialoceles previously reported.^37^ Our results demonstrated the presence of fat stranding in cases with or without infectious or inflammatory pathologies. Although not part of the objectives of this study, fat stranding does not appear to be a reliable feature in cases with infectious or inflammatory pathologies.

The present study also only identified the presence or absence of contrast enhancement but did not quantify the degree of enhancement. Between the cases with infectious or inflammatory pathologies and those without, the same proportion of cases (75%) displayed rim enhancement. It would be interesting to investigate whether the degree of enhancement is increased in cases with infectious or inflammatory pathologies.

The present study did not identify lymphadenomegaly even when an infectious or inflammatory pathology was present. Multiple regional lymph nodes of the head were assessed, each with individual reference ranges and scoring systems, which vary according to the size of the dog. As a result, the authors opted to assess the lymph node size qualitatively. Additionally, other features of lymphadenopathy, such as shape and attenuation, were not evaluated. We acknowledge that this is a limitation to our study and that any incidences of lymphadenopathy may be underreported. However, in the evaluation of all 22 cases, no significant lymph node enlargement was identified between the two reviewers, one of whom was an experienced ECVDI‐boarded radiologist.

As it was outside the scope of our study, the correlation between concurrent CT characteristics and identified pathologies was not calculated. Future studies are needed to investigate whether there is any association between specific CT features and pathologies.

Normal CT sialograms were observed with no visualized contrast leakage. These cases often involved either the sublingual or the mandibular gland. Anatomically, the sublingual and mandibular glands share a common capsule, and the corresponding ducts (major sublingual and mandibular ducts) are very closely associated and course adjacent to one another and open onto the sublingual caruncle.^41^ The sublingual and mandibular glands are surgically resected as a complex as standard when there is identifiable pathology in either of the glands.^42^ This close anatomical relationship was reflected in our results, allowing the identification of the affected gland to be made by exclusion in these cases. It is interesting that interobserver agreement for the detection of contrast leakage was poor. This may be related to the amorphous and random distribution of leaked contrast medium making it difficult to define a set standard.

The authors of a recent study suggested that identification of the specific diseased gland may not be clinically important, especially in cases involving the submandibular complex, since resection is en bloc regardless of the affected gland.^37^ However, the present study shows that CT sialography provides useful information for surgical planning, especially in cases where laterality could not be determined or if the extension of the sialocele was ambiguous. In these cases, identifying the gland of origin would help determine if a different or additional surgical approach had to be undertaken. This was demonstrated in case 5. Due to the dorsal extension of the sialocele (Figure 8), exclusion of parotid gland involvement was critical for surgical planning. Parotid sialoadenectomy is a more complex surgical procedure and requires a different approach than sialoadenectomy of the submandibular complex. Major vessels and other significant closely associated structures can also be delineated from the salivary gland apparatus prior to surgical exploration. Given further research, CT sialography could be of benefit for surgical planning and overall surgical management of sialoceles.

Our study found an overall moderate sensitivity of CT sialography in detecting surgically confirmed diseased glands. Individual sensitivities for the mandibular and sublingual glands were also moderate to high. Due to the small numbers involving the zygomatic and parotid glands, meaningful statistical analysis could not be performed to evaluate the sensitivity of CT sialography for these glands, which is a limitation of our study. Inclusion of more cases would provide greater confidence in the sensitivity of CT sialography, or the sublingual and mandibular glands could be considered a singular unit (the submandibular complex) given that they are resected en bloc. Establishment of a standardized, optimal CT sialography protocol could also improve the overall sensitivity of the technique.

Despite the varied level of experience in the surgery residents in training and supervising ECVS‐certified surgeons, cannulation of the salivary duct was successful in 21 of 22 cases, with only one nondiagnostic CT sialography study. This demonstrates that CT sialography is a feasible diagnostic technique that can be performed despite varying operator experience. Although not assessed in the present study, it would be interesting to investigate whether the level of operator experience would have any effect on the diagnostic quality of the CT sialography study and the overall sensitivity of this technique.

Although sialoceles were identified intraoperatively in all 22 dogs, only 14 were confirmed on histopathology. In the remaining eight dogs, pathologies such as sialadenitis were described as the main findings. This could be due to the difference between in vivo identification of a sialocele intraoperatively compared to histopathological examination of an *ex vivo* tissue specimen. The in vivo accumulation of saliva could have been lost in the fixation process, with analysis of only the affected salivary gland tissue, therefore resulting in a proportion of sialoceles that were not reported on histopathology. Additionally, in these eight cases, the confirmed pathologies are consistent with reported underlying causes of sialoceles, which would support the intraoperative identification of sialoceles.

The main limitation to this study was the retrospective nature and small number of cases. However, as discussed, salivary gland pathology is rare in dogs^1^, ^2^ and is often managed medically or surgically in first opinion practice without advanced diagnostic imaging. Even within the referral population presenting to the HfSA, only a proportion of the cases referred for suspected salivary gland disease received advanced imaging and/or surgical management due to a combination of patient, client, and clinician factors. These all contribute to the low number of patients who met the predetermined inclusion criteria. This limited population of patients could have affected the calculated sensitivity of this technique, as there could be an inherent selection bias of cases that could have influenced the CT sialography results. The authors recognize that sialoceles can be treated in first opinion practice without advanced imaging. However, in a proportion of cases, the swelling may be pronounced and nonlateralized. In these cases, identification of the affected gland may be challenging. Additionally, the actual extension of the sialocele may be more pronounced than its clinical appearance, as demonstrated in case 5 (Figure 8). Our study showed that CT sialography was subjectively a valuable technique to localize the affected salivary gland, and the authors found it useful, particularly in cases with a more complex presentation. Further studies comparing the surgical outcomes of cases receiving a CT sialography versus those that did not are needed to determine if CT sialography can be used to aid surgical planning and overall surgical management.

Our study population consisted mainly of medium‐ to large‐breed dogs, with the lowest weight of 8 kg. Salivary duct cannulation, especially for the submandibular gland complex, is challenging in very small dogs, which precluded their inclusion in our study. This is an area that would require further evaluation, and our findings may not represent CT sialography findings in smaller dogs. Another limitation is the introduction of bias. Although the cases were randomized and anonymized, both the diagnostic imaging resident in training (Y.L.T.) and reviewing radiologist (T.L.) were aware that surgically identified sialoceles were present in all cases. A third‐party arbiter could have been implemented in the event of any disagreement to minimize further bias. Nevertheless, there was good agreement on the detection of diseased salivary gland(s) by the reviewers as well as the diagnostic quality of the CT sialography studies. The lower agreement on the scoring of the CT sialography studies as well as the poor agreement on the detection of contrast leakage could be attributed to multiple factors, including difficulties interpreting finer or more subtle features, differences in experience of the reviewers and time spent evaluating the images. Additionally, due to the retrospective nature of the study, a standardized CT sialography protocol was not established, resulting in variation in the order of when the CT sialography study was performed. As a result, CT sialography and postcontrast findings could not be compared in more than half of the cases. This was because the authors felt that an objective assessment of the postcontrast studies could not be performed as contrast within the salivary apparatus would still be present following the sialograms performed. Although our study demonstrated that CT sialography provided additional information following the postcontrast study, this was in a small number of cases (n = 3). CT sialography identified the correct diseased gland more commonly (92.3%) based on “plain” CT sialography studies when CT sialography was performed before the postcontrast study compared to 62.5%, where diagnosis was based solely on postcontrast findings prior to CT sialography. However, our study did not demonstrate that performing the postcontrast study prior to CT sialography affected the final CT sialography diagnoses.

Nevertheless, the authors feel that CT sialography should be performed prior to the postcontrast study, as the presence of contrast enhancement of the adjacent structures or vasculature might hinder accurate interpretation of the CT sialography, where subtle sites of leakage could be missed. However, this is anecdotal based on the authors’ experience, and further studies comparing CT sialography with routine studies of the head and neck are needed to establish the diagnostic utility of CT sialography as well as to determine the optimal CT sialography protocol. Last, limited statistics were performed in our study due to the low number of cases. Due to the selection of the study population, cases without salivary gland disease were not included; therefore, the specificity of CT sialography could not be established.

Due to these limitations, future, prospective studies with a larger cohort and population size are needed to confirm our preliminary findings. Further comparison of CT sialography with routine studies of the head and neck is needed to establish the diagnostic utility of CT sialography.

## 5 CONCLUSION

In conclusion, salivary gland pathology is uncommon and rarely reported in dogs. Common CT and CT sialography findings were identified for this sample of dogs with surgically confirmed sialoceles. Computed tomography sialography identified the diseased salivary gland or duct with an overall sensitivity of 66.7%. Between the two reviewers, there was substantial agreement (κ = 0.70) on the identification of diseased gland(s) on CT sialography, substantial agreement (κ = 0.62) on the diagnostic quality evaluation, and no to slight agreement (к = 0.13) in the detection of contrast leakage. Future studies with larger cohorts and population sizes are needed to confirm our preliminary findings and to more definitively determine the utility of CT sialography in the surgical management of sialoceles.

## LIST OF AUTHOR CONTRIBUTIONS

### Category 1

(a) Conception and Design: Tan, Liuti, Marques, Schwarz
(b) Acquisition of Data: Tan, Liuti, Marques
(c) Analysis and Interpretation of Data: Tan, Liuti, Mitchell

### Category 2

(a) Drafting the Article: Tan, Liuti
(b) Revising the Article for Intellectual Content: Tan, Liuti, Marques, Schwarz, Mitchell

### Category 3

(a) Final Approval of the Completed Article: Tan, Liuti, Marques, Schwarz, Mitchell

### Category 4

(a) Agreement to be accountable for all aspects of the work in ensuring that questions related to the accuracy or integrity of any part of the work are appropriately investigated and resolved: Tan, Liuti, Marques, Schwarz, Mitchell

## REPORTING CHECKLIST DISCLOSURE

The authors followed the Strobe‐VET guidelines.

## CONFLICT OF INTEREST

The authors have declared no conflict of interest.

## Supporting information

Supplement 1: CT Sialography, Surgical and Histopathological Findings

Supplement 2
