# Supplementary material for: Clinical and CT sialography findings in 22 dogs with surgically confirmed sialoceles
Source: Vet Radiol Ultrasound. 2022 May 30;63(6):699–710. doi: 10.1111/vru.13104 (PMC9796823; doi:10.1111/vru.13104)
Supplement: Supplementary file 1 — Supplement 1: CT Sialography, Surgical and Histopathological Findings [file VRU-63-699-s001.docx]

Supplement 1: CT Sialography, Surgical and Histopathological Findings

| Case | Identified Gland(s) on CT Sialography | Identified Gland(s) in Surgery | Confirmed Histopathology | Diagnostic Quality | Presence of leakage/location |
| --- | --- | --- | --- | --- | --- |
| 1 | Mandibular (L) | Mandibular (L) | Sialocele | Good | Absent |
| 2 | Sublingual (L) | Sublingual, Mandibular (L) | Interstitial haemorrhage | Poor | Periductal |
| 3 | Mandibular (R) | Sublingual, Mandibular (R) | Sialocele | Poor | Periductal |
| 4 | Sublingual (L) | Sublingual (L) | Sialadenitis | Good | Absent |
| 5 | Sublingual (R) | Sublingual (R) | Abscessation secondary to sialocele | Good | Absent |
| 6 | Parotid (L) | Parotid (L) | Sialadenitis | Fair | Peri-glandular |
| 7 | No gland identified | Sublingual, Mandibular (L, R) | Sialocele, sialadenitis | Non-diagnostic | Absent |
| 8 | Mandibular (L) | Sublingual (L) | Sialocele, sialadenitis | Good | Absent |
| 9 | Sublingual (R) | Sublingual (R) | Sialocele, sialadenitis | Good | Absent |
| 10 | Mandibular (L) | Mandibular (L) | Sialocele, edema | Good | Peri-glandular, intra-sialocele |
| 11 | Sublingual (L) | Sublingual (L) | Sialadenitis | Good | Absent |
| 12 | Sublingual (L) | Sublingual (L) | Sialocele, sialadenitis | Good | Absent |
| 13 | Sublingual (R) | Sublingual (R) | Sialadenitis | Poor | Absent |
| 14 | Sublingual (L) | Sublingual (L) | Sialocele, sialadenitis | Good | Absent |
| 15 | Sublingual (L, R) | Sublingual (R, L), Mandibular (L) | Sialocele, cellulitis | Poor | Intra-sialocele (L, R) |
| 16 | Sublingual (L) | Sublingual (L) | Sialocele, sialadenitis | Poor | Intra-sialocele |
| 17 | Parotid (R) | Parotid (R) | Sialocele (chronic) | Good | Peri-glandular |
| 18 | Sublingual (R) | Sublingual (R) | Sialocele | Good | Absent |
| 19 | Zygomatic (L) | Zygomatic (L) | Sialadenitis | Good | Peri-glandular |
| 20 | Mandibular (L) | Sublingual, Mandibular, (L) | Sialadenitis | Good | Intra-sialocele, periductal |
| 21 | Sublingual (R) | Mandibular (R) | Sialocele, sialadenitis | Good | Intra-sialocele |
| 22 | Sublingual (R) | Sublingual (R) | Sialadenitis | Good | Intra-sialocele |
