# Supplementary material for: Clinical and CT sialography findings in 22 dogs with surgically confirmed sialoceles
Source: Vet Radiol Ultrasound. 2022 May 30;63(6):699–710. doi: 10.1111/vru.13104 (PMC9796823; doi:10.1111/vru.13104)
Supplement: Supplementary file 2 — Supplement 2 [file VRU-63-699-s002.docx]

| Case | Age  (Y) | Breed | Sex | Weight  (KG) | Clinical Signs | Size of Affected gland  (Normal, atrophied, enlarged) | Density (HU) | Local soft tissue appearance  (normal, fat stranding) | Accumulation of non-enhancing low density [0-20HU] fluid  (HU) | Capsule formation (present/  absent) and thickness (mm) | Regional Lymph nodes: (normal, enlarged) | Post-contrast: Absence/  presence, Location (Rim enhancement, core) |
| --- | --- | --- | --- | --- | --- | --- | --- | --- | --- | --- | --- | --- |
| 1 | 0.8 | Staffordshire Bull Terrier | FN | 17 | Submandibular swelling (L) | Normal | Normal soft tissue (49) | Normal | 4.85 | Present (1.5) | Normal | Present; rim enhancement |
| 2 | 0.8 | Crossbreed | MN | 15 | Swelling under the tongue | Normal | Normal soft tissue (42) | Normal | 14 | Present (1.4) | Normal | Present; rim enhancement |
| 3 | 1.7 | Miniature Dachshund | ME | 8 | Ventral neck swelling (R) | Normal | Normal soft tissue (55) | Fat stranding | 14.75 | Present (1.35) | Normal | Present; rim enhancement |
| 4 | 1.9 | Cocker Spaniel | MN | 10.9 | Intermandibular swelling | Normal | Normal soft tissue (65) | Fat stranding | 18.7 | Present (2.2) | Normal | Present; rim enhancement |
| 5 | 8.75 | Crossbreed | ME | 19 | Submandibular swelling (R) | Normal | Normal soft tissue (42) | Fat stranding | 20.6 | Present (3.5) | Normal | Present; rim enhancement |
| 6 | 4.7 | Cocker Spaniel | ME | 14 | Submandibular swelling (L) | Normal | Normal soft tissue (38) | Fat stranding | 6.2 | Present (1.1) | Normal | Present: rim enhancement |
| 7 | 11.5 | Crossbreed | MN | 29 | Large ventral midline neck and caudal mandibular swelling | NA | Normal soft tissue (65) | Fat stranding | 7 | Present (1.8) | Normal | Absent |
| 8 | 7.75 | Cocker Spaniel | MN | 17 | Submandibular mass (L) | Normal | Normal soft tissue (47) | Normal | 18 | Present (2) | Normal | Present; rim enhancement |
| 9 | 6.8 | Beagle | FN | 18 | Swelling under the tongue | Normal | Normal soft tissue (53) | Normal | 10 | Present (1.9) | Normal | Present; rim enhancement) |
| 10 | 0.3 | Labrador Retriever | FE | 15 | Submandibular swelling, wound discharging saliva (L) | Normal | Normal soft tissue (55) | Fat stranding | 6.15 | Present (3) | Normal | Present; rim enhancement |
| 11 | 3 | Siberian Husky | FN | 23 | Submandibular fluid-filled swelling | Normal | Normal soft tissue (59) | Normal | 6.85 | Present (1) | Normal | Present; rim enhancement |
| 12 | 4.75 | Border Collie | ME | 28.8 | Swelling under the tongue | Normal | Normal soft tissue (57) | Normal | 8.05 | Present (2) | Normal | Present; rim enhancement) |
| 13 | 2.5 | Northern Inuit | MN | 20 | Sublingual swelling | Normal | Normal soft tissue (57) | Normal | 23.5 | Present (1.35) | Normal | Present; rim enhancement) |
| 14 | 3.25 | Staffordshire Bull Terrier | ME | 17.7 | Swelling under the tongue (L) | Normal | Normal soft tissue (60) | Normal | 6.95 | Present (1.75) | Normal | Present; rim enhancement) |
| 15 | 0.75 | Siberian Husky | ME | 30 | Swelling at base of tongue (R) | Normal | Normal soft tissue  (L: 48; R 52) | Fat stranding (R)  Normal  (L) | 10.25 (L), 9.3 (R) | Present  (L:2 R:2.45) | Normal | Present; rim enhancement (bilateral) |
| 16 | 2.25 | Border Collie | FN | 17.6 | Fluid-filled swelling under the tongue (L) | Normal | Normal soft tissue (49) | Normal | 4.75 | Present (2.5) | Normal | Cannot be assessed |
| 17 | 12.4 | Cavalier King Charles Spaniel | MN | 13.1 | Swelling around angle of mandible (R) | Normal | Normal soft tissue (46) | Fat stranding | 10.6 | Present (3.8) | Normal | Absent |
| 18 | 4.3 | Border Collie | MN | 21.7 | Swelling at Submandibular (R), ventral neck and under the tongue | Normal | Normal soft tissue (53) | Normal | 11 | Present (1.25) | Normal | Present; rim enhancement |
| 19 | 10.2 | Labrador | FN | 41.8 | L exophthalmos | Enlarged | Normal soft tissue (58) | Normal | 21.5 | Absent | Normal | Absent |
| 20 | 4 | Collie | ME | 24 | Ventral neck swelling | Normal | Normal soft tissue (55) | Normal | 4.95 | Present (1.1) | Normal | Present; rim enhancement |
| 21 | 5.8 | Bearded Collie | FE | 20 | Ventral neck fluid-filled swelling | Normal | Normal soft tissue (63) | Normal | 10.25 | Present (1.6) | Normal | Present; rim enhancement |
| 22 | 1.2 | Lhasa Apso | MN | 10 | Intermandibular large fluid-filled swelling | Normal | Normal soft tissue (40) | Fat stranding | 11 | Absent | Normal | Absent |
